# Supplementary material for: Wide-Targeted Semi-Quantitative Analysis of Acidic Glycosphingolipids in Cell Lines and Urine to Develop Potential Screening Biomarkers for Renal Cell Carcinoma
Source: Int J Mol Sci. 2024 Apr 7;25(7):4098. doi: 10.3390/ijms25074098 (PMC11012862; doi:10.3390/ijms25074098)
Supplement: Supplementary file 1 [file ijms-25-04098-s001.zip › Figs_S_2.0.pptx]

## Slide 1
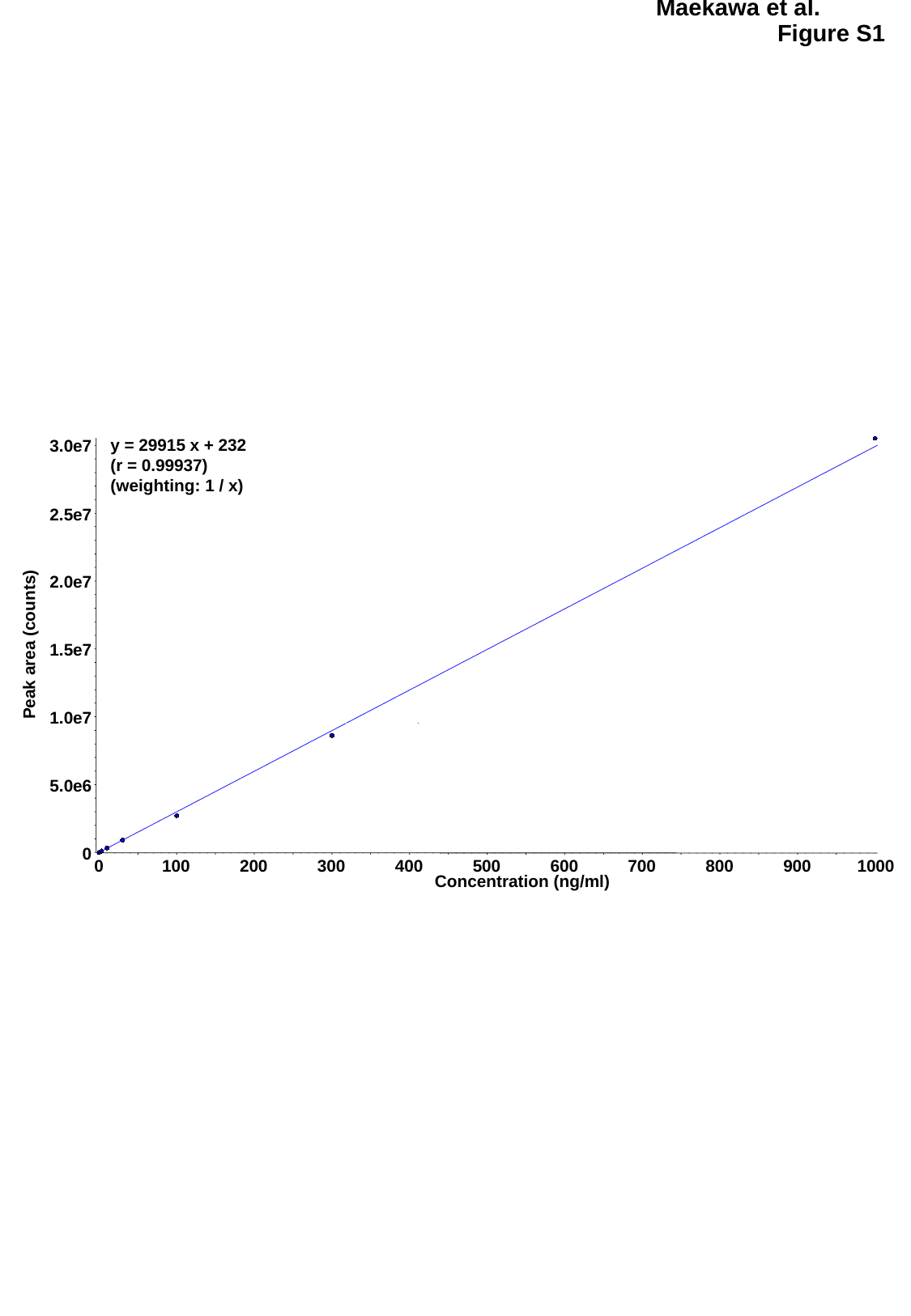

Maekawa et al.	Figure S1
y = 29915 x + 232
(r = 0.99937)
(weighting: 1 / x)
3.0e7
2.5e7
2.0e7
Peak area (counts)
1.5e7
1.0e7
5.0e6
0
0
100
200
300
400
500
600
700
800
900
1000
Concentration (ng/ml)

## Slide 2
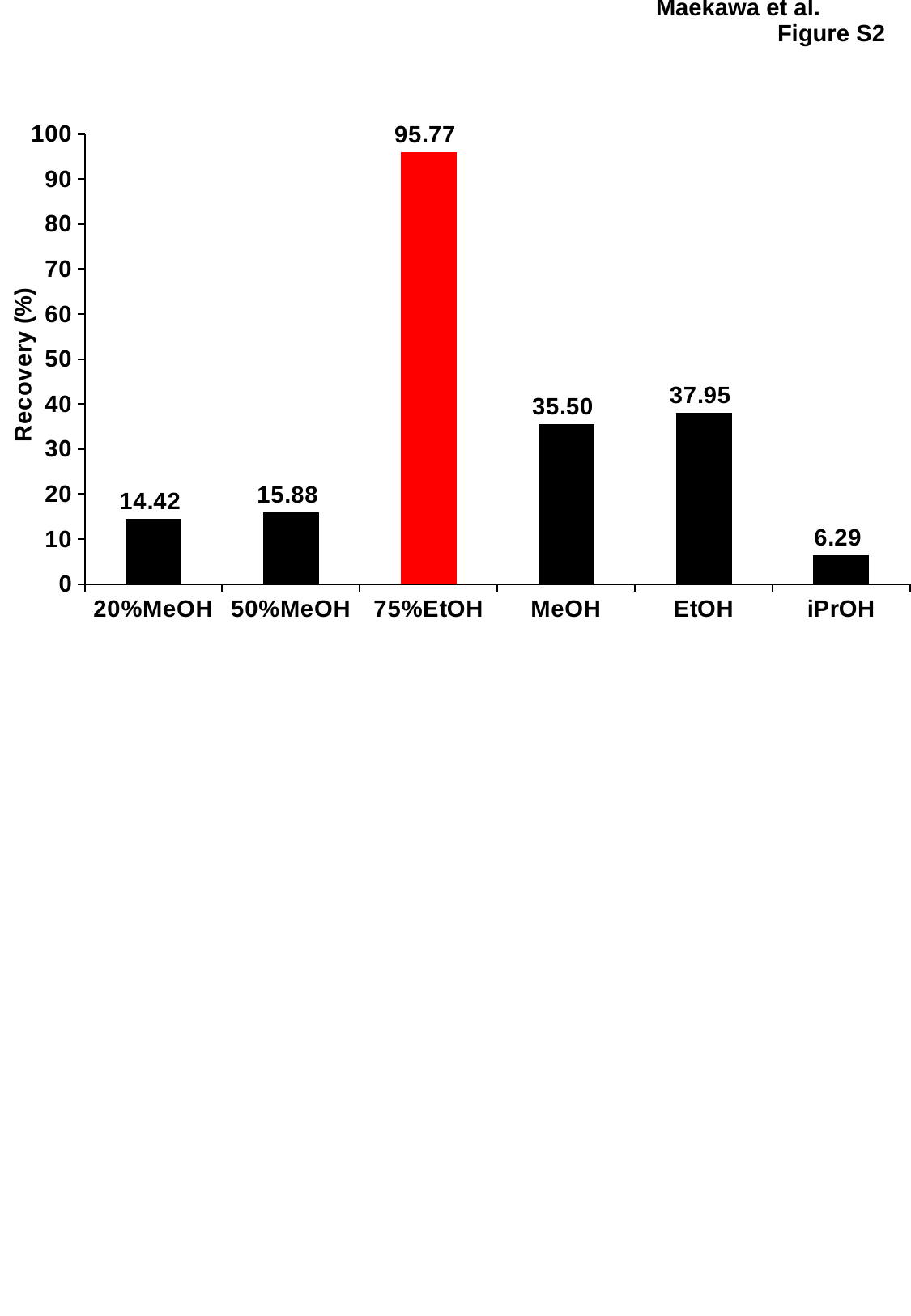

Maekawa et al.	Figure S2
### Chart
| Category | GD1a (d18:0) |
|---|---|
| 20%MeOH | 14.417425837097047 |
| 50%MeOH | 15.879062477256095 |
| 75%EtOH | 95.76837228281707 |
| MeOH | 35.50300379023716 |
| EtOH | 37.9525083691297 |
| iPrOH | 6.290121956587382 |

## Slide 3
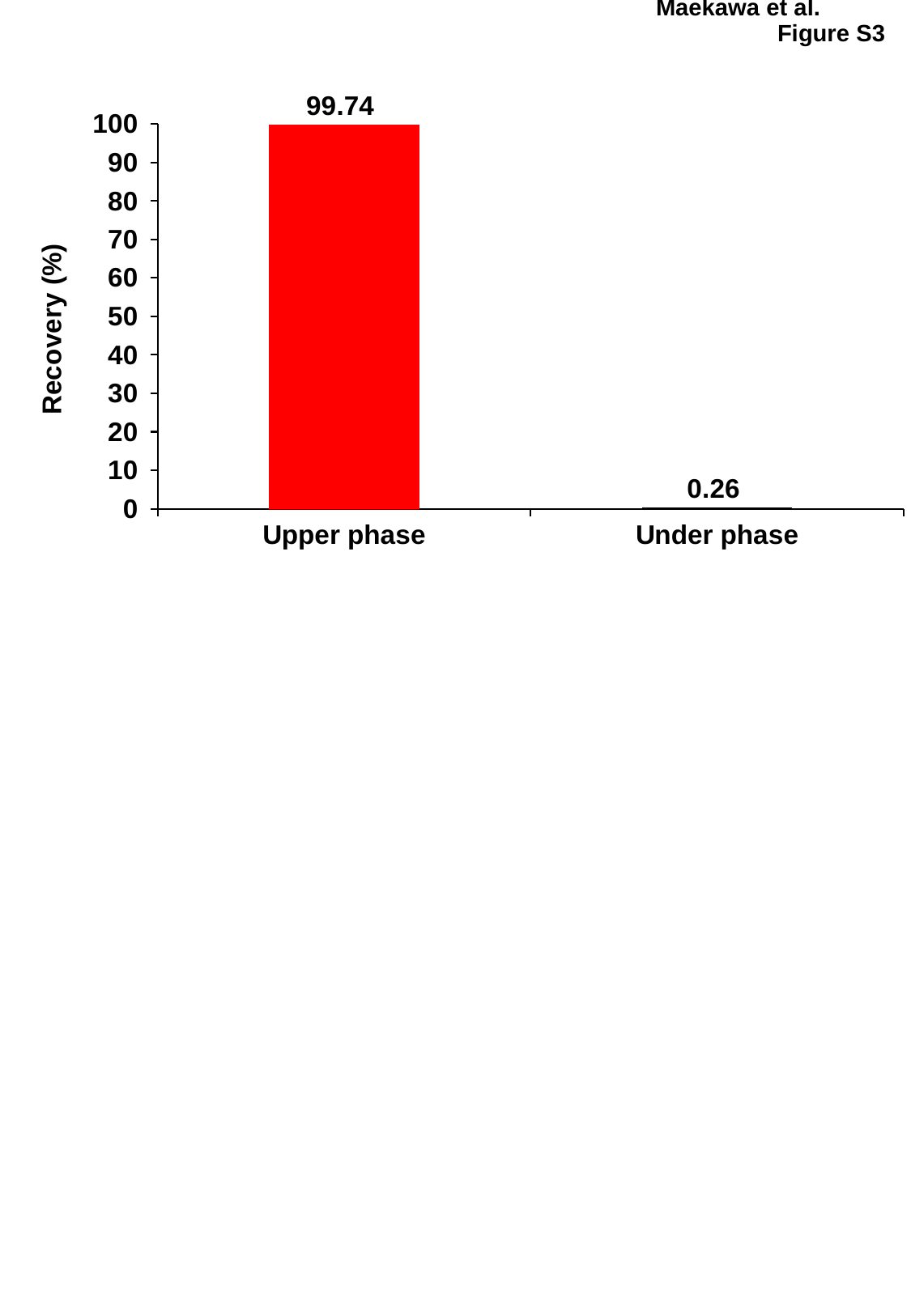

Maekawa et al.	Figure S3
### Chart
| Category | Ratio |
|---|---|
| Upper phase | 99.73613782564018 |
| Under phase | 0.26386217435982373 |
