# Supplementary material for: Wide-Targeted Semi-Quantitative Analysis of Acidic Glycosphingolipids in Cell Lines and Urine to Develop Potential Screening Biomarkers for Renal Cell Carcinoma
Source: Int J Mol Sci. 2024 Apr 7;25(7):4098. doi: 10.3390/ijms25074098 (PMC11012862; doi:10.3390/ijms25074098)
Supplement: Supplementary file 1 [file ijms-25-04098-s001.zip › TableS1_1.6.pdf]

Table S1. SRM transitions for wide-targeted GSLs analysis.

No.	Glycan	FA carbon (N)	Δ number (N)	Hex (N)	HexNAc (N)	NeuAc (N)	Fuc (N)	Q1 (*m/z*)	Q3 (*m/z*)
1	MSGb5	0	0	4	1	1	0	719.8	290.0
2	MSGb5	16	0	4	1	1	0	838.9	290.0
3	MSGb5	18	3	4	1	1	0	849.9	290.0
4	MSGb5	18	2	4	1	1	0	850.9	290.0
5	MSGb5	18	1	4	1	1	0	851.9	290.0
6	MSGb5	18	0	4	1	1	0	853.0	290.0
7	MSGb5	20	5	4	1	1	0	861.9	290.0
8	MSGb5	20	4	4	1	1	0	862.9	290.0
9	MSGb5	20	3	4	1	1	0	863.9	290.0
10	MSGb5	20	2	4	1	1	0	865.0	290.0
11	MSGb5	20	1	4	1	1	0	866.0	290.0
12	MSGb5	20	0	4	1	1	0	867.0	290.0
13	MSGb5	22	6	4	1	1	0	874.9	290.0
14	MSGb5	22	5	4	1	1	0	875.9	290.0
15	MSGb5	22	4	4	1	1	0	877.0	290.0
16	MSGb5	22	3	4	1	1	0	878.0	290.0
17	MSGb5	22	2	4	1	1	0	879.0	290.0
18	MSGb5	22	1	4	1	1	0	880.0	290.0
19	MSGb5	22	0	4	1	1	0	881.0	290.0
20	MSGb5	24	1	4	1	1	0	894.0	290.0

No.	Glycan	FA carbon (N)	Δ number (N)	Hex (N)	HexNAc (N)	NeuAc (N)	Fuc (N)	Q1 (*m/z*)	Q3 (*m/z*)
21	MSGb5	24	0	4	1	1	0	895.0	290.0
22	MSGb5	26	0	4	1	1	0	909.0	290.0
23	MSGb5	28	0	4	1	1	0	923.0	290.0
24	MSGb5	30	0	4	1	1	0	937.1	290.0
25	DSGb5	0	0	4	1	2	0	865.4	290.0
26	DSGb5	16	0	4	1	2	0	984.5	290.0
27	DSGb5	18	3	4	1	2	0	995.5	290.0
28	DSGb5	18	2	4	1	2	0	996.5	290.0
29	DSGb5	18	1	4	1	2	0	997.5	290.0
30	DSGb5	18	0	4	1	2	0	998.5	290.0
31	DSGb5	20	5	4	1	2	0	1007.5	290.0
32	DSGb5	20	4	4	1	2	0	1008.5	290.0
33	DSGb5	20	3	4	1	2	0	1009.5	290.0
34	DSGb5	20	2	4	1	2	0	1010.5	290.0
35	DSGb5	20	1	4	1	2	0	1011.5	290.0
36	DSGb5	20	0	4	1	2	0	1012.5	290.0
37	DSGb5	22	6	4	1	2	0	1020.5	290.0
38	DSGb5	22	5	4	1	2	0	1021.5	290.0
39	DSGb5	22	4	4	1	2	0	1022.5	290.0
40	DSGb5	22	3	4	1	2	0	1023.5	290.0
41	DSGb5	22	2	4	1	2	0	1024.5	290.0

No.	Glycan	FA carbon (N)	Δ number (N)	Hex (N)	HexNAc (N)	NeuAc (N)	Fuc (N)	Q1 (*m/z*)	Q3 (*m/z*)
42	DSGb5	22	1	4	1	2	0	1025.5	290.0
43	DSGb5	22	0	4	1	2	0	1026.5	290.0
44	DSGb5	24	1	4	1	2	0	1039.5	290.0
45	DSGb5	24	0	4	1	2	0	1040.6	290.0
46	DSGb5	26	0	4	1	2	0	1054.6	290.0
47	DSGb5	28	0	4	1	2	0	1068.6	290.0
48	DSGb5	30	0	4	1	2	0	1082.6	290.0
49	GM3	0	0	2	0	1	0	913.5	290.0
50	GM3	16	0	2	0	1	0	1151.7	290.0
51	GM3	18	3	2	0	1	0	1173.7	290.0
52	GM3	18	2	2	0	1	0	1175.7	290.0
53	GM3	18	1	2	0	1	0	1177.7	290.0
54	GM3	18	0	2	0	1	0	1179.7	290.0
55	GM3	20	5	2	0	1	0	1197.7	290.0
56	GM3	20	4	2	0	1	0	1199.7	290.0
57	GM3	20	3	2	0	1	0	1201.7	290.0
58	GM3	20	2	2	0	1	0	1203.7	290.0
59	GM3	20	1	2	0	1	0	1205.8	290.0
60	GM3	20	0	2	0	1	0	1207.8	290.0
61	GM3	22	6	2	0	1	0	1223.7	290.0
62	GM3	22	5	2	0	1	0	1225.7	290.0

No.	Glycan	FA carbon (N)	Δ number (N)	Hex (N)	HexNAc (N)	NeuAc (N)	Fuc (N)	Q1 (*m/z*)	Q3 (*m/z*)
63	GM3	22	4	2	0	1	0	1227.7	290.0
64	GM3	22	3	2	0	1	0	1229.8	290.0
65	GM3	22	2	2	0	1	0	1231.8	290.0
66	GM3	22	1	2	0	1	0	1233.8	290.0
67	GM3	22	0	2	0	1	0	1235.8	290.0
68	GM3	24	1	2	0	1	0	1261.8	290.0
69	GM3	24	0	2	0	1	0	1263.8	290.0
70	GM3	26	0	2	0	1	0	1291.9	290.0
71	GM3	28	0	2	0	1	0	1319.9	290.0
72	GM3	30	0	2	0	1	0	1347.9	290.0
73	GM2	0	0	2	1	1	0	1116.6	290.0
74	GM2	16	0	2	1	1	0	1354.8	290.0
75	GM2	18	3	2	1	1	0	1376.8	290.0
76	GM2	18	2	2	1	1	0	1378.8	290.0
77	GM2	18	1	2	1	1	0	1380.8	290.0
78	GM2	18	0	2	1	1	0	1382.8	290.0
79	GM2	20	5	2	1	1	0	1400.8	290.0
80	GM2	20	4	2	1	1	0	1402.8	290.0
81	GM2	20	3	2	1	1	0	1404.8	290.0
82	GM2	20	2	2	1	1	0	1406.8	290.0
83	GM2	20	1	2	1	1	0	1408.8	290.0

No.	Glycan	FA carbon (N)	Δ number (N)	Hex (N)	HexNAc (N)	NeuAc (N)	Fuc (N)	Q1 (*m/z*)	Q3 (*m/z*)
84	GM2	20	0	2	1	1	0	1410.8	290.0
85	GM2	22	6	2	1	1	0	1426.8	290.0
86	GM2	22	5	2	1	1	0	1428.8	290.0
87	GM2	22	4	2	1	1	0	1430.8	290.0
88	GM2	22	3	2	1	1	0	1432.8	290.0
89	GM2	22	2	2	1	1	0	1434.8	290.0
90	GM2	22	1	2	1	1	0	1436.9	290.0
91	GM2	22	0	2	1	1	0	1438.9	290.0
92	GM2	24	1	2	1	1	0	1464.9	290.0
93	GM2	24	0	2	1	1	0	1466.9	290.0
94	GM2	26	0	2	1	1	0	1494.9	290.0
95	GM2	28	0	2	1	1	0	1523.0	290.0
96	GM2	30	0	2	1	1	0	1551.0	290.0
97	GM1/DUPAN-2	0	0	3	1	1	0	1278.6	290.0
98	GM1/DUPAN-2	16	0	3	1	1	0	1516.8	290.0
99	GM1/DUPAN-2	18	3	3	1	1	0	1538.8	290.0
100	GM1/DUPAN-2	18	2	3	1	1	0	1540.8	290.0
101	GM1/DUPAN-2	18	1	3	1	1	0	1542.9	290.0
102	GM1/DUPAN-2	18	0	3	1	1	0	1544.9	290.0
103	GM1/DUPAN-2	20	5	3	1	1	0	1562.8	290.0
104	GM1/DUPAN-2	20	4	3	1	1	0	1564.8	290.0

No.	Glycan	FA carbon (N)	Δ number (N)	Hex (N)	HexNAc (N)	NeuAc (N)	Fuc (N)	Q1 (*m/z*)	Q3 (*m/z*)
105	GM1/DUPAN-2	20	3	3	1	1	0	1566.9	290.0
106	GM1/DUPAN-2	20	2	3	1	1	0	1568.9	290.0
107	GM1/DUPAN-2	20	1	3	1	1	0	1570.9	290.0
108	GM1/DUPAN-2	20	0	3	1	1	0	1572.9	290.0
109	GM1/DUPAN-2	22	6	3	1	1	0	1588.8	290.0
110	GM1/DUPAN-2	22	5	3	1	1	0	1590.9	290.0
111	GM1/DUPAN-2	22	4	3	1	1	0	1592.9	290.0
112	GM1/DUPAN-2	22	3	3	1	1	0	1594.9	290.0
113	GM1/DUPAN-2	22	2	3	1	1	0	1596.9	290.0
114	GM1/DUPAN-2	22	1	3	1	1	0	1598.9	290.0
115	GM1/DUPAN-2	22	0	3	1	1	0	1600.9	290.0
116	GM1/DUPAN-2	24	1	3	1	1	0	1626.9	290.0
117	GM1/DUPAN-2	24	0	3	1	1	0	1629.0	290.0
118	GM1/DUPAN-2	26	0	3	1	1	0	1657.0	290.0
119	GM1/DUPAN-2	28	0	3	1	1	0	1685.0	290.0
120	GM1/DUPAN-2	30	0	3	1	1	0	1713.1	290.0
121	GD1a/DSLc4	0	0	3	1	2	0	784.3	290.0
122	GD1a/DSLc4	16	0	3	1	2	0	903.5	290.0
123	GD1a/DSLc4	18	3	3	1	2	0	914.5	290.0
124	GD1a/DSLc4	18	2	3	1	2	0	915.5	290.0
125	GD1a/DSLc4	18	1	3	1	2	0	916.5	290.0

No.	Glycan	FA carbon (N)	Δ number (N)	Hex (N)	HexNAc (N)	NeuAc (N)	Fuc (N)	Q1 (*m/z*)	Q3 (*m/z*)
126	GD1a/DSLc4	18	0	3	1	2	0	917.5	290.0
127	GD1a/DSLc4	20	5	3	1	2	0	926.5	290.0
128	GD1a/DSLc4	20	4	3	1	2	0	927.5	290.0
129	GD1a/DSLc4	20	3	3	1	2	0	928.5	290.0
130	GD1a/DSLc4	20	2	3	1	2	0	929.5	290.0
131	GD1a/DSLc4	20	1	3	1	2	0	930.5	290.0
132	GD1a/DSLc4	20	0	3	1	2	0	931.5	290.0
133	GD1a/DSLc4	22	6	3	1	2	0	939.5	290.0
134	GD1a/DSLc4	22	5	3	1	2	0	940.5	290.0
135	GD1a/DSLc4	22	4	3	1	2	0	941.5	290.0
136	GD1a/DSLc4	22	3	3	1	2	0	942.5	290.0
137	GD1a/DSLc4	22	2	3	1	2	0	943.5	290.0
138	GD1a/DSLc4	22	1	3	1	2	0	944.5	290.0
139	GD1a/DSLc4	22	0	3	1	2	0	945.5	290.0
140	GD1a/DSLc4	24	1	3	1	2	0	958.5	290.0
141	GD1a/DSLc4	24	0	3	1	2	0	959.5	290.0
142	GD1a/DSLc4	26	0	3	1	2	0	973.5	290.0
143	GD1a/DSLc4	28	0	3	1	2	0	987.6	290.0
144	GD1a/DSLc4	30	0	3	1	2	0	1001.6	290.0
145	GalNAcDSLc4	0	0	3	2	2	0	885.9	290.0
146	GalNAcDSLc4	16	0	3	2	2	0	1005.0	290.0

No.	Glycan	FA carbon (N)	Δ number (N)	Hex (N)	HexNAc (N)	NeuAc (N)	Fuc (N)	Q1 (*m/z*)	Q3 (*m/z*)
147	GalNAcDSLc4	18	3	3	2	2	0	1016.0	290.0
148	GalNAcDSLc4	18	2	3	2	2	0	1017.0	290.0
149	GalNAcDSLc4	18	1	3	2	2	0	1018.0	290.0
150	GalNAcDSLc4	18	0	3	2	2	0	1019.0	290.0
151	GalNAcDSLc4	20	5	3	2	2	0	1028.0	290.0
152	GalNAcDSLc4	20	4	3	2	2	0	1029.0	290.0
153	GalNAcDSLc4	20	3	3	2	2	0	1030.0	290.0
154	GalNAcDSLc4	20	2	3	2	2	0	1031.0	290.0
155	GalNAcDSLc4	20	1	3	2	2	0	1032.0	290.0
156	GalNAcDSLc4	20	0	3	2	2	0	1033.0	290.0
157	GalNAcDSLc4	22	6	3	2	2	0	1041.0	290.0
158	GalNAcDSLc4	22	5	3	2	2	0	1042.0	290.0
159	GalNAcDSLc4	22	4	3	2	2	0	1043.0	290.0
160	GalNAcDSLc4	22	3	3	2	2	0	1044.0	290.0
161	GalNAcDSLc4	22	2	3	2	2	0	1045.0	290.0
162	GalNAcDSLc4	22	1	3	2	2	0	1046.0	290.0
163	GalNAcDSLc4	22	0	3	2	2	0	1047.0	290.0
164	GalNAcDSLc4	24	1	3	2	2	0	1060.1	290.0
165	GalNAcDSLc4	24	0	3	2	2	0	1061.1	290.0
166	GalNAcDSLc4	26	0	3	2	2	0	1075.1	290.0
167	GalNAcDSLc4	28	0	3	2	2	0	1089.1	290.0

No.	Glycan	FA carbon (N)	Δ number (N)	Hex (N)	HexNAc (N)	NeuAc (N)	Fuc (N)	Q1 (*m/z*)	Q3 (*m/z*)
168	GalNAcDSLc4	30	0	3	2	2	0	1103.1	290.0
169	CA19-9	0	0	3	1	1	1	1424.7	290.0
170	CA19-9	16	0	3	1	1	1	1662.9	290.0
171	CA19-9	18	3	3	1	1	1	1684.9	290.0
172	CA19-9	18	2	3	1	1	1	1686.9	290.0
173	CA19-9	18	1	3	1	1	1	1688.9	290.0
174	CA19-9	18	0	3	1	1	1	1690.9	290.0
175	CA19-9	20	5	3	1	1	1	1708.9	290.0
176	CA19-9	20	4	3	1	1	1	1710.9	290.0
177	CA19-9	20	3	3	1	1	1	1712.9	290.0
178	CA19-9	20	2	3	1	1	1	1714.9	290.0
179	CA19-9	20	1	3	1	1	1	1716.9	290.0
180	CA19-9	20	0	3	1	1	1	1719.0	290.0
181	CA19-9	22	6	3	1	1	1	1734.9	290.0
182	CA19-9	22	5	3	1	1	1	1736.9	290.0
183	CA19-9	22	4	3	1	1	1	1738.9	290.0
184	CA19-9	22	3	3	1	1	1	1740.9	290.0
185	CA19-9	22	2	3	1	1	1	1743.0	290.0
186	CA19-9	22	1	3	1	1	1	1745.0	290.0
187	CA19-9	22	0	3	1	1	1	1747.0	290.0
188	CA19-9	24	1	3	1	1	1	1773.0	290.0

No.	Glycan	FA carbon (N)	Δ number (N)	Hex (N)	HexNAc (N)	NeuAc (N)	Fuc (N)	Q1 (*m/z*)	Q3 (*m/z*)
189	CA19-9	24	0	3	1	1	1	1775.0	290.0
190	CA19-9	26	0	3	1	1	1	1803.1	290.0
191	CA19-9	28	0	3	1	1	1	1831.1	290.0
192	CA19-9	30	0	3	1	1	1	1859.1	290.0
193	Speculated 1	0	0	5	1	2	0	946.4	290.0
194	Speculated 1	16	0	5	1	2	0	1065.5	290.0
195	Speculated 1	18	3	5	1	2	0	1076.5	290.0
196	Speculated 1	18	2	5	1	2	0	1077.5	290.0
197	Speculated 1	18	1	5	1	2	0	1078.5	290.0
198	Speculated 1	18	0	5	1	2	0	1079.5	290.0
199	Speculated 1	20	5	5	1	2	0	1088.5	290.0
200	Speculated 1	20	4	5	1	2	0	1089.5	290.0
201	Speculated 1	20	3	5	1	2	0	1090.5	290.0
202	Speculated 1	20	2	5	1	2	0	1091.5	290.0
203	Speculated 1	20	1	5	1	2	0	1092.5	290.0
204	Speculated 1	20	0	5	1	2	0	1093.5	290.0
205	Speculated 1	22	6	5	1	2	0	1101.5	290.0
206	Speculated 1	22	5	5	1	2	0	1102.5	290.0
207	Speculated 1	22	4	5	1	2	0	1103.5	290.0
208	Speculated 1	22	3	5	1	2	0	1104.5	290.0
209	Speculated 1	22	2	5	1	2	0	1105.5	290.0

No.	Glycan	FA carbon (N)	Δ number (N)	Hex (N)	HexNAc (N)	NeuAc (N)	Fuc (N)	Q1 (*m/z*)	Q3 (*m/z*)
210	Speculated 1	22	1	5	1	2	0	1106.6	290.0
211	Speculated 1	22	0	5	1	2	0	1107.6	290.0
212	Speculated 1	24	1	5	1	2	0	1120.6	290.0
213	Speculated 1	24	0	5	1	2	0	1121.6	290.0
214	Speculated 1	26	0	5	1	2	0	1135.6	290.0
215	Speculated 1	28	0	5	1	2	0	1149.6	290.0
216	Speculated 1	30	0	5	1	2	0	1163.6	290.0
217	Speculated 2	0	0	4	2	2	0	966.9	290.0
218	Speculated 2	16	0	4	2	2	0	1086.0	290.0
219	Speculated 2	18	3	4	2	2	0	1097.0	290.0
220	Speculated 2	18	2	4	2	2	0	1098.0	290.0
221	Speculated 2	18	1	4	2	2	0	1099.0	290.0
222	Speculated 2	18	0	4	2	2	0	1100.0	290.0
223	Speculated 2	20	5	4	2	2	0	1109.0	290.0
224	Speculated 2	20	4	4	2	2	0	1110.0	290.0
225	Speculated 2	20	3	4	2	2	0	1111.0	290.0
226	Speculated 2	20	2	4	2	2	0	1112.0	290.0
227	Speculated 2	20	1	4	2	2	0	1113.1	290.0
228	Speculated 2	20	0	4	2	2	0	1114.1	290.0
229	Speculated 2	22	6	4	2	2	0	1122.0	290.0
230	Speculated 2	22	5	4	2	2	0	1123.0	290.0

No.	Glycan	FA carbon (N)	Δ number (N)	Hex (N)	HexNAc (N)	NeuAc (N)	Fuc (N)	Q1 (*m/z*)	Q3 (*m/z*)
231	Speculated 2	22	4	4	2	2	0	1124.0	290.0
232	Speculated 2	22	3	4	2	2	0	1125.1	290.0
233	Speculated 2	22	2	4	2	2	0	1126.1	290.0
234	Speculated 2	22	1	4	2	2	0	1127.1	290.0
235	Speculated 2	22	0	4	2	2	0	1128.1	290.0
236	Speculated 2	24	1	4	2	2	0	1141.1	290.0
237	Speculated 2	24	0	4	2	2	0	1142.1	290.0
238	Speculated 2	26	0	4	2	2	0	1156.1	290.0
239	Speculated 2	28	0	4	2	2	0	1170.1	290.0
240	Speculated 2	30	0	4	2	2	0	1184.1	290.0
