# Supplementary material for: Wide-Targeted Semi-Quantitative Analysis of Acidic Glycosphingolipids in Cell Lines and Urine to Develop Potential Screening Biomarkers for Renal Cell Carcinoma
Source: Int J Mol Sci. 2024 Apr 7;25(7):4098. doi: 10.3390/ijms25074098 (PMC11012862; doi:10.3390/ijms25074098)
Supplement: Supplementary file 1 [file ijms-25-04098-s001.zip › TableS2_1.6.pdf]

Table S2 MS/MS and ion source parameters for GSLs analysis.

(a) MS/MS parameters for wide targeted GSL analysis

Compound	The number of NeuAc	DP (V)	EP (V)	CE (V)	CXP (V)
MSGb5<br>GM3<br>GM2<br>GM1/DUPAN-2<br>CA19-9	1	-65	-6	-80	-9
DSGb5<br>GD1a/DSLc4<br>GalNAcDSLc4<br>Speculated 1<br>Speculated 2	2	-65	-6	-50	-9

CE, collision energy; CXP, collision cell exit potential; DP, declustering potential; EP, entrance potential.

(b) Ion source parameters for wide targeted GSL analysis

CUR (psi)	CAD (unit)	ISV (V)	TEM (ºC)	GS1 (psi)	GS2 (psi)
10	12	-4500	500	60	60

CAD, collision gas; CUR, curtain gas; GS, ion source gas; ISV, ionspray voltage; TEM, turbo gas temperature.
