# Supplementary material for: Wide-Targeted Semi-Quantitative Analysis of Acidic Glycosphingolipids in Cell Lines and Urine to Develop Potential Screening Biomarkers for Renal Cell Carcinoma
Source: Int J Mol Sci. 2024 Apr 7;25(7):4098. doi: 10.3390/ijms25074098 (PMC11012862; doi:10.3390/ijms25074098)
Supplement: Supplementary file 1 [file ijms-25-04098-s001.zip › TableS3_1.6.pdf]

Table S3 Gradient programs for wide-targeted GSLs analysis.

Time (min)	B (%)	Flow rate (mL/min)
0	40	0.4
40	100	0.4
45	100	0.4
