# Supplementary material for: Wide-Targeted Semi-Quantitative Analysis of Acidic Glycosphingolipids in Cell Lines and Urine to Develop Potential Screening Biomarkers for Renal Cell Carcinoma
Source: Int J Mol Sci. 2024 Apr 7;25(7):4098. doi: 10.3390/ijms25074098 (PMC11012862; doi:10.3390/ijms25074098)
Supplement: Supplementary file 1 [file ijms-25-04098-s001.zip › TableS4_2.0.pdf]

Table S4 Matrix factor of GD1a (d18:1/18:0) using the ACHN cell medium.

	The peak area in 10 ng/mL Standard solution (counts)	The peak area in ACHN Cell medium (counts)	The peak area of 10 ng/mL Standard solution spiked in ACHN Cell medium (counts)	Matrix factor (%)
GD1a (d18:1/18:0)	1157865	264968	1486623	106
