# Supplementary material for: Wide-Targeted Semi-Quantitative Analysis of Acidic Glycosphingolipids in Cell Lines and Urine to Develop Potential Screening Biomarkers for Renal Cell Carcinoma
Source: Int J Mol Sci. 2024 Apr 7;25(7):4098. doi: 10.3390/ijms25074098 (PMC11012862; doi:10.3390/ijms25074098)
Supplement: Supplementary file 1 [file ijms-25-04098-s001.zip › TableS5_2.0.pdf]

Table S5. GSL analysis results for cell lines using wide-targeted GSL analysis.

(a) The peak areas and the ratio of GSL peaks in HK-2 cells and ACHN cells.

No.	Components	**Q1/Q3 (*m/z*)**	**Cell HK-2 (counts/10^6cells)**	**Cell ACHN (counts/10^6cells))**	**Ratio ACHN to HK-2 (%)**
1	CA19-9 (d18:1/0:0)	1424.7 / 290.1	0	16828	**N.A.**
2	CA19-9 (d18:1/16:0)	1662.9 / 290.1	938	961	**103%**
3	CA19-9 (d18:1/18:0)	1690.9 / 290.1	0	1682	**N.A.**
4	CA19-9 (d18:1/18:1)	1688.9 / 290.1	0	2404	**N.A.**
5	CA19-9 (d18:1/18:2)	1686.9 / 290.1	0	1444	**N.A.**
6	CA19-9 (d18:1/18:3)	1684.9 / 290.1	0	961	**N.A.**
7	CA19-9 (d18:1/20:0)	1719.0 / 290.1	0	961	**N.A.**
8	CA19-9 (d18:1/20:1)	1716.9 / 290.1	0	1199	**N.A.**
9	CA19-9 (d18:1/20:2)	1714.9 / 290.1	0	961	**N.A.**
10	CA19-9 (d18:1/20:3)	1712.9 / 290.1	0	481	**N.A.**
11	CA19-9 (d18:1/20:4)	1710.9 / 290.1	0	2164	**N.A.**
12	CA19-9 (d18:1/20:5)	1708.9 / 290.1	1866	6008	**322%**
13	CA19-9 (d18:1/22:0)	1747.0 / 290.1	0	1202	**N.A.**
14	CA19-9 (d18:1/22:1)	1745.0 / 290.1	0	961	**N.A.**
15	CA19-9 (d18:1/22:2)	1743.0 / 290.1	0	1201	**N.A.**
16	CA19-9 (d18:1/22:3)	1740.9 / 290.1	0	0	**N.A.**
17	CA19-9 (d18:1/22:4)	1738.9 / 290.1	0	961	**N.A.**
18	CA19-9 (d18:1/22:5)	1736.9 / 290.1	0	961	**N.A.**
19	CA19-9 (d18:1/22:6)	1734.9 / 290.1	0	1923	**N.A.**

No.	Components	**Q1/Q3** **(*m/z*)**	**Cell HK-2** **(counts/10^6cells)**	**Cell ACHN** **(counts/10^6cells))**	**Ratio ACHN to HK-2** **(%)**
20	CA19-9 (d18:1/24:0)	1775.0 / 290.1	0	1201	**N.A.**
21	CA19-9 (d18:1/24:1)	1773.0 / 290.1	0	3124	**N.A.**
22	CA19-9 (d18:1/26:0)	1803.1 / 290.1	0	2163	**N.A.**
23	CA19-9 (d18:1/28:0)	1831.1 / 290.1	0	721	**N.A.**
24	CA19-9 (d18:1/30:0)	1859.1 / 290.1	0	0	**N.A.**
25	DSGb5 (d18:1/0:0) 1	865.4 / 290.1	4184	465241	**11119%**
26	DSGb5 (d18:1/0:0) 2	865.4 / 290.1	104872	1028253	**980%**
27	DSGb5 (d18:1/0:0) 3	865.4 / 290.1	10727	671044	**6256%**
28	DSGb5 (d18:1/16:0)	984.5 / 290.1	22144663	56032660	**253%**
29	DSGb5 (d18:1/18:0)	998.5 / 290.1	1867691	11033419	**591%**
30	DSGb5 (d18:1/18:1)	997.5 / 290.1	67872	632994	**933%**
31	DSGb5 (d18:1/18:2)	996.5 / 290.1	460656	909345	**197%**
32	DSGb5 (d18:1/18:3)	995.5 / 290.1	962763	1886512	**196%**
33	DSGb5 (d18:1/20:0)	1012.5 / 290.1	922063	6382497	**692%**
34	DSGb5 (d18:1/20:1)	1011.5 / 290.1	271704	1276628	**470%**
35	DSGb5 (d18:1/20:2)	1010.5 / 290.1	0	548261	**N.A.**
36	DSGb5 (d18:1/20:3)	1009.5 / 290.1	101816	837621	**823%**
37	DSGb5 (d18:1/20:4)	1008.5 / 290.1	2104	71994	**3422%**
38	DSGb5 (d18:1/20:5)	1007.5 / 290.1	83673	148404	**177%**
39	DSGb5 (d18:1/22:0)	1026.5 / 290.1	4144129	30136140	**727%**

No.	Components	**Q1/Q3 (*m/z*)**	**Cell HK-2 (counts/10^6cells)**	**Cell ACHN (counts/10^6cells))**	**Ratio ACHN to HK-2 (%)**
40	DSGb5 (d18:1/22:1)	1025.5 / 290.1	1813228	12480051	**688%**
41	DSGb5 (d18:1/22:2)	1024.5 / 290.1	286602	806247	**281%**
42	DSGb5 (d18:1/22:3)	1023.5 / 290.1	52591	323719	**616%**
43	DSGb5 (d18:1/22:4)	1022.5 / 290.1	16038	101428	**632%**
44	DSGb5 (d18:1/22:5)	1021.5 / 290.1	8977	120438	**1342%**
45	DSGb5 (d18:1/22:6)	1020.5 / 290.1	51499	362595	**704%**
46	DSGb5 (d18:1/24:0) 1	1040.6 / 290.1	6376444	25571015	**401%**
47	DSGb5 (d18:1/24:0) 2	1040.6 / 290.1	6191572	39235054	**634%**
48	DSGb5 (d18:1/24:1) 1	1039.5 / 290.1	1682279	6328533	**376%**
49	DSGb5 (d18:1/24:1) 2	1039.5 / 290.1	14504290	52454845	**362%**
50	DSGb5 (d18:1/26:0) 1	1054.6 / 290.1	276755	2365213	**855%**
51	DSGb5 (d18:1/26:0) 2	1054.6 / 290.1	132632	2117141	**1596%**
52	DSGb5 (d18:1/28:0)	1068.6 / 290.1	12923	31340	**243%**
53	DSGb5 (d18:1/30:0)	1082.6 / 290.1	4189	36594	**874%**
54	GalNAcDSLc4, RM2 (d18:1/0:0)	885.9 / 290.1	87550	267537	**306%**
55	GalNAcDSLc4, RM2 (d18:1/16:0)	1005.0 / 290.1	70772	815625	**1152%**
56	GalNAcDSLc4, RM2 (d18:1/18:0)	1019.0 / 290.1	73498	380358	**518%**
57	GalNAcDSLc4, RM2 (d18:1/18:1)	1018.0 / 290.1	11246	182343	**1621%**
58	GalNAcDSLc4, RM2 (d18:1/18:2)	1017.0 / 290.1	25616	323785	**1264%**
59	GalNAcDSLc4, RM2 (d18:1/18:3)	1016.0 / 290.1	9279	111055	**1197%**

No.	Components	**Q1/Q3 (*m/z*)**	**Cell HK-2 (counts/10^6cells)**	**Cell ACHN (counts/10^6cells))**	**Ratio ACHN to HK-2 (%)**
60	GalNAcDSLc4, RM2 (d18:1/20:0) 1	1033.0 / 290.1	1313089	5926848	**451%**
61	GalNAcDSLc4, RM2 (d18:1/20:0) 2	1033.0 / 290.1	423960	1899270	**448%**
62	GalNAcDSLc4, RM2 (d18:1/20:1)	1032.0 / 290.1	706396	2878341	**407%**
63	GalNAcDSLc4, RM2 (d18:1/20:2)	1031.0 / 290.1	47194	330206	**700%**
64	GalNAcDSLc4, RM2 (d18:1/20:3)	1030.0 / 290.1	26600	79439	**299%**
65	GalNAcDSLc4, RM2 (d18:1/20:4)	1029.0 / 290.1	91283	784508	**859%**
66	GalNAcDSLc4, RM2 (d18:1/20:5)	1028.0 / 290.1	849502	7437006	**875%**
67	GalNAcDSLc4, RM2 (d18:1/22:0) 1	1047.0 / 290.1	552120	2062564	**374%**
68	GalNAcDSLc4, RM2 (d18:1/22:0) 2	1047.0 / 290.1	108528	404395	**373%**
69	GalNAcDSLc4, RM2 (d18:1/22:1)	1046.0 / 290.1	269271	986679	**366%**
70	GalNAcDSLc4, RM2 (d18:1/22:2)	1045.0 / 290.1	110172	323958	**294%**
71	GalNAcDSLc4, RM2 (d18:1/22:3)	1044.0 / 290.1	84372	185761	**220%**
72	GalNAcDSLc4, RM2 (d18:1/22:4)	1043.0 / 290.1	127300	1091295	**857%**
73	GalNAcDSLc4, RM2 (d18:1/22:5) 1	1042.0 / 290.1	326087	1446688	**444%**
74	GalNAcDSLc4, RM2 (d18:1/22:5) 2	1042.0 / 290.1	1363818	10931696	**802%**
75	GalNAcDSLc4, RM2 (d18:1/22:6) 1	1041.0 / 290.1	3568511	15904736	**446%**
76	GalNAcDSLc4, RM2 (d18:1/22:6) 2	1041.0 / 290.1	5579133	38101626	**683%**
77	GalNAcDSLc4, RM2 (d18:1/24:0)	1061.1 / 290.1	31616	462449	**1463%**
78	GalNAcDSLc4, RM2 (d18:1/24:1)	1060.1 / 290.1	33867	635953	**1878%**
79	GalNAcDSLc4, RM2 (d18:1/26:0)	1075.1 / 290.1	9807	24522	**250%**

No.	Components	**Q1/Q3 (*m/z*)**	**Cell HK-2 (counts/10^6cells)**	**Cell ACHN (counts/10^6cells))**	**Ratio ACHN to HK-2 (%)**
80	GalNAcDSLc4, RM2 (d18:1/28:0)	1089.1 / 290.1	10554	30558	**290%**
81	GalNAcDSLc4, RM2 (d18:1/30:0)	1103.1 / 290.1	30855	245794	**797%**
82	GD1a (d18:1/0:0)	784.3 / 290.1	0	585343	**N.A.**
83	GD1a (d18:1/16:0)	903.5 / 290.1	18405655	63951269	**347%**
84	GD1a (d18:1/18:0)	917.5 / 290.1	2078299	8939450	**430%**
85	GD1a (d18:1/18:1) 1	916.5 / 290.1	364657	939568	**258%**
86	GD1a (d18:1/18:1) 2	916.5 / 290.1	161013	603723	**375%**
87	GD1a (d18:1/18:1) 3	916.5 / 290.1	135446	697406	**515%**
88	GD1a (d18:1/18:2) 1	915.5 / 290.1	187718	593506	**316%**
89	GD1a (d18:1/18:2) 2	915.5 / 290.1	329476	1765555	**536%**
90	GD1a (d18:1/18:3)	914.5 / 290.1	424212	1578221	**372%**
91	GD1a (d18:1/20:0)	931.5 / 290.1	859977	5628198	**654%**
92	GD1a (d18:1/20:1)	930.5 / 290.1	206430	1157868	**561%**
93	GD1a (d18:1/20:2)	929.5 / 290.1	32666	163966	**502%**
94	GD1a (d18:1/20:3)	928.5 / 290.1	61276	284039	**464%**
95	GD1a (d18:1/20:4)	927.5 / 290.1	5261	226438	**4304%**
96	GD1a (d18:1/20:5)	926.5 / 290.1	59344	107529	**181%**
97	GD1a (d18:1/22:0) 1	945.5 / 290.1	466512	4747651	**1018%**
98	GD1a (d18:1/22:0) 2	945.5 / 290.1	3493311	30233435	**865%**
99	GD1a (d18:1/22:1) 1	944.5 / 290.1	0	979304	**N.A.**

No.	Components	**Q1/Q3** **(*m/z*)**	**Cell HK-2** **(counts/10^6cells)**	**Cell ACHN** **(counts/10^6cells))**	**Ratio ACHN to HK-2** **(%)**
100	GD1a (d18:1/22:1) 2	944.5 / 290.1	1245983	11346404	**911%**
101	GD1a (d18:1/22:1) 3	944.5 / 290.1	132189	1749622	**1324%**
102	GD1a (d18:1/22:2)	943.5 / 290.1	2335	2819946	**120750%**
103	GD1a (d18:1/22:3)	942.5 / 290.1	14365	590109	**4108%**
104	GD1a (d18:1/22:4)	941.5 / 290.1	109298	1465852	**1341%**
105	GD1a (d18:1/22:5)	940.5 / 290.1	22296	611749	**2744%**
106	GD1a (d18:1/22:6)	939.5 / 290.1	33738	361889	**1073%**
107	GD1a (d18:1/24:0) 1	959.5 / 290.1	4123877	19292435	**468%**
108	GD1a (d18:1/24:0) 2	959.5 / 290.1	4842411	40530077	**837%**
109	GD1a (d18:1/24:1) 1	958.5 / 290.1	820287	4621854	**563%**
110	GD1a (d18:1/24:1) 2	958.5 / 290.1	10029584	47487629	**473%**
111	GD1a (d18:1/24:1) 3	958.5 / 290.1	138961	1902813	**1369%**
112	GD1a (d18:1/26:0) 1	973.5 / 290.1	199011	2337680	**1175%**
113	GD1a (d18:1/26:0) 2	973.5 / 290.1	107490	1929916	**1795%**
114	GD1a (d18:1/28:0)	987.6 / 290.1	104921	263451	**251%**
115	GD1a (d18:1/30:0)	1001.6 / 290.1	956	221441	**23161%**
116	GM1 (d18:1/0:0)	1278.6 / 290.1	1377	15673	**1138%**
117	GM1 (d18:1/16:0)	1516.8 / 290.1	41752	160289	**384%**
118	GM1 (d18:1/18:0)	1544.9 / 290.1	5627	14658	**261%**
119	GM1 (d18:1/18:1)	1542.9 / 290.1	0	4646	**N.A.**

No.	Components	**Q1/Q3 (*m/z*)**	**Cell HK-2 (counts/10^6cells)**	**Cell ACHN (counts/10^6cells))**	**Ratio ACHN to HK-2 (%)**
120	GM1 (d18:1/18:2)	1540.8 / 290.1	0	6746	**N.A.**
121	GM1 (d18:1/18:3)	1538.8 / 290.1	938	9600	**1024%**
122	GM1 (d18:1/20:0)	1572.9 / 290.1	0	4325	**N.A.**
123	GM1 (d18:1/20:1)	1570.9 / 290.1	1406	7993	**568%**
124	GM1 (d18:1/20:2)	1568.9 / 290.1	0	3146	**N.A.**
125	GM1 (d18:1/20:3)	1566.9 / 290.1	0	1923	**N.A.**
126	GM1 (d18:1/20:4)	1564.8 / 290.1	0	721	**N.A.**
127	GM1 (d18:1/20:5)	1562.8 / 290.1	0	721	**N.A.**
128	GM1 (d18:1/22:0)	1600.9 / 290.1	0	12496	**N.A.**
129	GM1 (d18:1/22:1)	1598.9 / 290.1	0	4085	**N.A.**
130	GM1 (d18:1/22:2)	1596.9 / 290.1	938	4085	**436%**
131	GM1 (d18:1/22:3)	1594.9 / 290.1	0	1842	**N.A.**
132	GM1 (d18:1/22:4)	1592.9 / 290.1	0	721	**N.A.**
133	GM1 (d18:1/22:5)	1590.9 / 290.1	0	491	**N.A.**
134	GM1 (d18:1/22:6)	1588.8 / 290.1	0	721	**N.A.**
135	GM1 (d18:1/24:0)	1629.0 / 290.1	14209	51796	**365%**
136	GM1 (d18:1/24:1)	1626.9 / 290.1	0	21736	**N.A.**
137	GM1 (d18:1/26:0)	1657.0 / 290.1	1406	3124	**222%**
138	GM1 (d18:1/28:0)	1685.0 / 290.1	0	721	**N.A.**
139	GM1 (d18:1/30:0)	1713.1 / 290.1	0	1442	**N.A.**

No.	Components	**Q1/Q3 (*m/z*)**	**Cell HK-2 (counts/10^6cells)**	**Cell ACHN (counts/10^6cells))**	**Ratio ACHN to HK-2 (%)**
140	GM2 (d18:1/0:0)	1116.6 / 290.1	2334	96782	**4146%**
141	GM2 (d18:1/16:0)	1354.8 / 290.1	1017367	7018688	**690%**
142	GM2 (d18:1/18:0)	1382.8 / 290.1	42670	687558	**1611%**
143	GM2 (d18:1/18:1)	1380.8 / 290.1	17123	68758	**402%**
144	GM2 (d18:1/18:2)	1378.8 / 290.1	1411	7630	**541%**
145	GM2 (d18:1/18:3)	1376.8 / 290.1	4221	17972	**426%**
146	GM2 (d18:1/20:0)	1410.8 / 290.1	7033	183937	**2615%**
147	GM2 (d18:1/20:1)	1408.8 / 290.1	0	37246	**N.A.**
148	GM2 (d18:1/20:2)	1406.8 / 290.1	1406	5284	**376%**
149	GM2 (d18:1/20:3)	1404.8 / 290.1	0	3604	**N.A.**
150	GM2 (d18:1/20:4)	1402.8 / 290.1	0	3593	**N.A.**
151	GM2 (d18:1/20:5)	1400.8 / 290.1	0	3416	**N.A.**
152	GM2 (d18:1/22:0)	1438.9 / 290.1	22037	384682	**1746%**
153	GM2 (d18:1/22:1)	1436.9 / 290.1	11722	125981	**1075%**
154	GM2 (d18:1/22:2)	1434.8 / 290.1	0	9413	**N.A.**
155	GM2 (d18:1/22:3)	1432.8 / 290.1	0	1474	**N.A.**
156	GM2 (d18:1/22:4)	1430.8 / 290.1	938	4351	**464%**
157	GM2 (d18:1/22:5)	1428.8 / 290.1	4220	13655	**324%**
158	GM2 (d18:1/22:6)	1426.8 / 290.1	0	13811	**N.A.**
159	GM2 (d18:1/24:0)	1466.9 / 290.1	101289	1114595	**1100%**

No.	Components	**Q1/Q3 (*m/z*)**	**Cell HK-2 (counts/10^6cells)**	**Cell ACHN (counts/10^6cells))**	**Ratio ACHN to HK-2 (%)**
160	GM2 (d18:1/24:1)	1464.9 / 290.1	51576	319244	**619%**
161	GM2 (d18:1/26:0)	1494.9 / 290.1	1406	28117	**2000%**
162	GM2 (d18:1/28:0)	1523.0 / 290.1	2344	1659	**71%**
163	GM2 (d18:1/30:0)	1551.0 / 290.1	4689	13464	**287%**
164	GM3 (d18:1/0:0)	913.5 / 290.1	3224	331646	**10286%**
165	GM3 (d18:1/16:0)	1151.7 / 290.1	12745039	16748086	**131%**
166	GM3 (d18:1/18:0)	1179.7 / 290.1	716656	2486179	**347%**
167	GM3 (d18:1/18:1)	1177.7 / 290.1	196011	514072	**262%**
168	GM3 (d18:1/18:2)	1175.7 / 290.1	1435	15648	**1090%**
169	GM3 (d18:1/18:3)	1173.7 / 290.1	11815	8279	**70%**
170	GM3 (d18:1/20:0)	1207.8 / 290.1	122934	406867	**331%**
171	GM3 (d18:1/20:1)	1205.8 / 290.1	22512	82907	**368%**
172	GM3 (d18:1/20:2)	1203.7 / 290.1	11718	19490	**166%**
173	GM3 (d18:1/20:3)	1201.7 / 290.1	938	4538	**484%**
174	GM3 (d18:1/20:4)	1199.7 / 290.1	2506	18229	**727%**
175	GM3 (d18:1/20:5)	1197.7 / 290.1	5304	23597	**445%**
176	GM3 (d18:1/22:0)	1235.8 / 290.1	302041	966394	**320%**
177	GM3 (d18:1/22:1)	1233.8 / 290.1	74557	280873	**377%**
178	GM3 (d18:1/22:2)	1231.8 / 290.1	12660	24958	**197%**
179	GM3 (d18:1/22:3)	1229.8 / 290.1	2832	10648	**376%**

No.	Components	**Q1/Q3 (*m/z*)**	**Cell HK-2 (counts/10^6cells)**	**Cell ACHN (counts/10^6cells))**	**Ratio ACHN to HK-2 (%)**
180	GM3 (d18:1/22:4)	1227.7 / 290.1	0	3063	**N.A.**
181	GM3 (d18:1/22:5)	1225.7 / 290.1	1789	10889	**609%**
182	GM3 (d18:1/22:6)	1223.7 / 290.1	5637	28727	**510%**
183	GM3 (d18:1/24:0)	1263.8 / 290.1	735145	1721588	**234%**
184	GM3 (d18:1/24:1)	1261.8 / 290.1	370051	639977	**173%**
185	GM3 (d18:1/26:0)	1291.9 / 290.1	15004	64413	**429%**
186	GM3 (d18:1/28:0)	1319.9 / 290.1	938	2312	**247%**
187	GM3 (d18:1/30:0)	1347.9 / 290.1	0	3407	**N.A.**
188	MSGb5 (d18:1/0:0)	719.8 / 290.1	47839	587159	**1227%**
189	MSGb5 (d18:1/16:0) 1	838.9 / 290.1	6680	6418501	**96078%**
190	MSGb5 (d18:1/16:0) 2	838.9 / 290.1	11831866	32037533	**271%**
191	MSGb5 (d18:1/18:0) 1	853.0 / 290.1	44554	27911024	**62646%**
192	MSGb5 (d18:1/18:0) 2	853.0 / 290.1	831800	4863876	**585%**
193	MSGb5 (d18:1/18:1) 1	851.9 / 290.1	16895	6255382	**37025%**
194	MSGb5 (d18:1/18:1) 2	851.9 / 290.1	154122	1549533	**1005%**
195	MSGb5 (d18:1/18:2)	850.9 / 290.1	39321	915796	**2329%**
196	MSGb5 (d18:1/18:3)	849.9 / 290.1	35017	246373	**704%**
197	MSGb5 (d18:1/20:0) 1	867.0 / 290.1	40289	3904110	**9690%**
198	MSGb5 (d18:1/20:0) 2	867.0 / 290.1	483168	2099817	**435%**
199	MSGb5 (d18:1/20:1)	866.0 / 290.1	8901	1228163	**13798%**

No.	Components	**Q1/Q3 (*m/z*)**	**Cell HK-2 (counts/10^6cells)**	**Cell ACHN (counts/10^6cells))**	**Ratio ACHN to HK-2 (%)**
200	MSGb5 (d18:1/20:2) 1	865.0 / 290.1	5073	586395	**11559%**
201	MSGb5 (d18:1/20:2) 2	865.0 / 290.1	123167	1416729	**1150%**
202	MSGb5 (d18:1/20:3) 1	863.9 / 290.1	48776	1116397	**2289%**
203	MSGb5 (d18:1/20:3) 2	863.9 / 290.1	83893	1104251	**1316%**
204	MSGb5 (d18:1/20:4)	862.9 / 290.1	2726	626903	**23000%**
205	MSGb5 (d18:1/20:5)	861.9 / 290.1	1765	536379	**30396%**
206	MSGb5 (d18:1/22:0) 1	881.0 / 290.1	172884	830130	**480%**
207	MSGb5 (d18:1/22:0) 2	881.0 / 290.1	2045582	11971671	**585%**
208	MSGb5 (d18:1/22:1) 1	880.0 / 290.1	546557	2743441	**502%**
209	MSGb5 (d18:1/22:1) 2	880.0 / 290.1	90540	564956	**624%**
210	MSGb5 (d18:1/22:2)	879.0 / 290.1	161123	1596421	**991%**
211	MSGb5 (d18:1/22:3) 1	878.0 / 290.1	356137	1843731	**518%**
212	MSGb5 (d18:1/22:3) 2	878.0 / 290.1	110234	1203334	**1092%**
213	MSGb5 (d18:1/22:4)	877.0 / 290.1	233745	1383616	**592%**
214	MSGb5 (d18:1/22:5)	875.9 / 290.1	45662	162351	**356%**
215	MSGb5 (d18:1/22:6)	874.9 / 290.1	36200	146284	**404%**
216	MSGb5 (d18:1/24:0) 1	895.0 / 290.1	1778389	5653999	**318%**
217	MSGb5 (d18:1/24:0) 2	895.0 / 290.1	5164683	19827504	**384%**
218	MSGb5 (d18:1/24:1) 1	894.0 / 290.1	273456	613257	**224%**
219	MSGb5 (d18:1/24:1) 2	894.0 / 290.1	4851418	15395102	**317%**

No.	Components	**Q1/Q3** **(*m/z*)**	**Cell HK-2** **(counts/10^6cells)**	**Cell ACHN** **(counts/10^6cells))**	**Ratio ACHN to HK-2** **(%)**
220	MSGb5 (d18:1/24:1) 3	894.0 / 290.1	350804	1282352	**366%**
221	MSGb5 (d18:1/26:0)	909.0 / 290.1	190239	935727	**492%**
222	MSGb5 (d18:1/28:0)	923.0 / 290.1	74505	270926	**364%**
223	MSGb5 (d18:1/30:0)	937.1 / 290.1	15209	141356	**929%**
224	Speculated 1 (d18:1/0:0) 1	946.4 / 290.1	2139109	18592528	**869%**
225	Speculated 1 (d18:1/0:0) 2	946.4 / 290.1	44089	1131576	**2567%**
226	Speculated 1 (d18:1/16:0)	1065.5 / 290.1	5046	76232	**1511%**
227	Speculated 1 (d18:1/18:0)	1079.5 / 290.1	8766	12704	**145%**
228	Speculated 1 (d18:1/18:1)	1078.5 / 290.1	17176	43652	**254%**
229	Speculated 1 (d18:1/18:2)	1077.5 / 290.1	28606	165501	**579%**
230	Speculated 1 (d18:1/18:3)	1076.5 / 290.1	17945	83326	**464%**
231	Speculated 1 (d18:1/20:0)	1093.5 / 290.1	6076	35521	**585%**
232	Speculated 1 (d18:1/20:1)	1092.5 / 290.1	18852	296286	**1572%**
233	Speculated 1 (d18:1/20:2)	1091.5 / 290.1	37902	1148574	**3030%**
234	Speculated 1 (d18:1/20:3)	1090.5 / 290.1	33544	587144	**1750%**
235	Speculated 1 (d18:1/20:4)	1089.5 / 290.1	11030	92121	**835%**
236	Speculated 1 (d18:1/20:5)	1088.5 / 290.1	9796	19863	**203%**
237	Speculated 1 (d18:1/22:0)	1107.6 / 290.1	1502	37710	**2510%**
238	Speculated 1 (d18:1/22:1)	1106.6 / 290.1	12049	413985	**3436%**
239	Speculated 1 (d18:1/22:2)	1105.5 / 290.1	31710	2051850	**6471%**

No.	Components	**Q1/Q3 (*m/z*)**	**Cell HK-2 (counts/10^6cells)**	**Cell ACHN (counts/10^6cells))**	**Ratio ACHN to HK-2 (%)**
240	Speculated 1 (d18:1/22:3) 1	1104.5 / 290.1	103860	2351849	**2264%**
241	Speculated 1 (d18:1/22:3) 2	1104.5 / 290.1	13488	920341	**6824%**
242	Speculated 1 (d18:1/22:4)	1103.5 / 290.1	55980	1093191	**1953%**
243	Speculated 1 (d18:1/22:5)	1102.5 / 290.1	15339	94423	**616%**
244	Speculated 1 (d18:1/22:6)	1101.5 / 290.1	4321	31931	**739%**
245	Speculated 1 (d18:1/24:0)	1121.6 / 290.1	9565	44143	**462%**
246	Speculated 1 (d18:1/24:1)	1120.6 / 290.1	954	24989	**2620%**
247	Speculated 1 (d18:1/26:0)	1135.6 / 290.1	0	16646	**N.A.**
248	Speculated 1 (d18:1/28:0)	1149.6 / 290.1	346349	1107839	**320%**
249	Speculated 1 (d18:1/30:0)	1163.6 / 290.1	0	29210	**N.A.**
250	Speculated 2 (d18:1/0:0) 1	966.9 / 290.1	360535	1416874	**393%**
251	Speculated 2 (d18:1/0:0) 2	966.9 / 290.1	219002	1632364	**745%**
252	Speculated 2 (d18:1/16:0)	1086.0 / 290.1	272240	199296	**73%**
253	Speculated 2 (d18:1/18:0)	1100.0 / 290.1	10029	36641	**365%**
254	Speculated 2 (d18:1/18:1)	1099.0 / 290.1	9606	82533	**859%**
255	Speculated 2 (d18:1/18:2)	1098.0 / 290.1	7320	186518	**2548%**
256	Speculated 2 (d18:1/18:3)	1097.0 / 290.1	31777	176329	**555%**
257	Speculated 2 (d18:1/20:0)	1114.1 / 290.1	5483	46614	**850%**
258	Speculated 2 (d18:1/20:1)	1113.1 / 290.1	5490	242070	**4409%**
259	Speculated 2 (d18:1/20:2)	1112.0 / 290.1	13878	595346	**4290%**

No.	Components	**Q1/Q3**<br>**(*m/z*)**	**Cell HK-2**<br>**(counts/10^6cells)**	**Cell ACHN**<br>**(counts/10^6cells))**	**Ratio ACHN to HK-2**<br>**(%)**
260	Speculated 2 (d18:1/20:3)	1111.0 / 290.1	6623	82558	**1247%**
261	Speculated 2 (d18:1/20:4)	1110.0 / 290.1	2209	19843	**898%**
262	Speculated 2 (d18:1/20:5)	1109.0 / 290.1	4911	29139	**593%**
263	Speculated 2 (d18:1/22:0)	1128.1 / 290.1	957	71162	**7433%**
264	Speculated 2 (d18:1/22:1)	1127.1 / 290.1	1767	423058	**23941%**
265	Speculated 2 (d18:1/22:2)	1126.1 / 290.1	3756	854411	**22747%**
266	Speculated 2 (d18:1/22:3) 1	1125.1 / 290.1	10602	685479	**6465%**
267	Speculated 2 (d18:1/22:3) 2	1125.1 / 290.1	127476	469542	**368%**
268	Speculated 2 (d18:1/22:4)	1124.0 / 290.1	476840	1439654	**302%**
269	Speculated 2 (d18:1/22:5)	1123.0 / 290.1	310127	1033985	**333%**
270	Speculated 2 (d18:1/22:6)	1122.0 / 290.1	12995	30650	**236%**
271	Speculated 2 (d18:1/24:0)	1142.1 / 290.1	1784	69653	**3904%**
272	Speculated 2 (d18:1/24:1)	1141.1 / 290.1	16466	126398	**768%**
273	Speculated 2 (d18:1/26:0)	1156.1 / 290.1	18937	129495	**684%**
274	Speculated 2 (d18:1/28:0)	1170.1 / 290.1	100501	219078	**218%**
275	Speculated 2 (d18:1/30:0)	1184.1 / 290.1	2313	12238	**529%**
276	Total		179734772	845115961	**470%**

N.A., not applicable.

(b) The peak areas and the ratio of GSL peaks in medium of HK-2 cells and ACHN cells.

No.	Components	**Q1/Q3 (*m/z*)**	**Medium HK-2 (counts)**	**Medium ACHN (counts)**	**Ratio ACHN to HK-2 (%)**
1	CA19-9 (d18:1/0:0)	1424.7 / 290.1	0	0	#DIV/0!
2	CA19-9 (d18:1/16:0)	1662.9 / 290.1	0	0	#DIV/0!
3	CA19-9 (d18:1/18:0)	1690.9 / 290.1	0	0	#DIV/0!
4	CA19-9 (d18:1/18:1)	1688.9 / 290.1	0	0	#DIV/0!
5	CA19-9 (d18:1/18:2)	1686.9 / 290.1	0	0	#DIV/0!
6	CA19-9 (d18:1/18:3)	1684.9 / 290.1	0	0	#DIV/0!
7	CA19-9 (d18:1/20:0)	1719.0 / 290.1	0	0	#DIV/0!
8	CA19-9 (d18:1/20:1)	1716.9 / 290.1	0	0	#DIV/0!
9	CA19-9 (d18:1/20:2)	1714.9 / 290.1	0	769	#DIV/0!
10	CA19-9 (d18:1/20:3)	1712.9 / 290.1	0	0	#DIV/0!
11	CA19-9 (d18:1/20:4)	1710.9 / 290.1	0	0	#DIV/0!
12	CA19-9 (d18:1/20:5)	1708.9 / 290.1	0	0	#DIV/0!
13	CA19-9 (d18:1/22:0)	1747.0 / 290.1	0	0	#DIV/0!
14	CA19-9 (d18:1/22:1)	1745.0 / 290.1	0	0	#DIV/0!
15	CA19-9 (d18:1/22:2)	1743.0 / 290.1	0	0	#DIV/0!
16	CA19-9 (d18:1/22:3)	1740.9 / 290.1	0	0	#DIV/0!
17	CA19-9 (d18:1/22:4)	1738.9 / 290.1	0	0	#DIV/0!
18	CA19-9 (d18:1/22:5)	1736.9 / 290.1	0	0	#DIV/0!
19	CA19-9 (d18:1/22:6)	1734.9 / 290.1	0	0	#DIV/0!
20	CA19-9 (d18:1/24:0)	1775.0 / 290.1	0	0	#DIV/0!

No.	Components	**Q1/Q3 (*m/z*)**	**Medium HK-2 (counts)**	**Medium ACHN (counts)**	**Ratio ACHN to HK-2 (%)**
21	CA19-9 (d18:1/24:1)	1773.0 / 290.1	0	0	#DIV/0!
22	CA19-9 (d18:1/26:0)	1803.1 / 290.1	0	0	#DIV/0!
23	CA19-9 (d18:1/28:0)	1831.1 / 290.1	0	0	#DIV/0!
24	CA19-9 (d18:1/30:0)	1859.1 / 290.1	0	0	#DIV/0!
25	DSGb5 (d18:1/0:0) 1	865.4 / 290.1	3453	2112	61%
26	DSGb5 (d18:1/0:0) 2	865.4 / 290.1	8423	7072	84%
27	DSGb5 (d18:1/0:0) 3	865.4 / 290.1	1941	4012	207%
28	DSGb5 (d18:1/16:0)	984.5 / 290.1	997593	4577916	459%
29	DSGb5 (d18:1/18:0)	998.5 / 290.1	77100	135718	176%
30	DSGb5 (d18:1/18:1)	997.5 / 290.1	6648	8447	127%
31	DSGb5 (d18:1/18:2)	996.5 / 290.1	11824	99546	842%
32	DSGb5 (d18:1/18:3)	995.5 / 290.1	38128	245233	643%
33	DSGb5 (d18:1/20:0)	1012.5 / 290.1	5545	20777	375%
34	DSGb5 (d18:1/20:1)	1011.5 / 290.1	8762	15663	179%
35	DSGb5 (d18:1/20:2)	1010.5 / 290.1	1880	1102	59%
36	DSGb5 (d18:1/20:3)	1009.5 / 290.1	3841	7292	190%
37	DSGb5 (d18:1/20:4)	1008.5 / 290.1	2691	3586	133%
38	DSGb5 (d18:1/20:5)	1007.5 / 290.1	7484	20674	276%
39	DSGb5 (d18:1/22:0)	1026.5 / 290.1	15354	55602	362%
40	DSGb5 (d18:1/22:1)	1025.5 / 290.1	14997	26336	176%

No.	Components	**Q1/Q3 (*m/z*)**	**Medium HK-2 (counts)**	**Medium ACHN (counts)**	**Ratio ACHN to HK-2 (%)**
41	DSGb5 (d18:1/22:2)	1024.5 / 290.1	1158	12032	1039%
42	DSGb5 (d18:1/22:3)	1023.5 / 290.1	5373	3463	64%
43	DSGb5 (d18:1/22:4)	1022.5 / 290.1	900	1893	210%
44	DSGb5 (d18:1/22:5)	1021.5 / 290.1	0	1841	#DIV/0!
45	DSGb5 (d18:1/22:6)	1020.5 / 290.1	1396	3845	275%
46	DSGb5 (d18:1/24:0) 1	1040.6 / 290.1	31937	45330	142%
47	DSGb5 (d18:1/24:0) 2	1040.6 / 290.1	13829	25295	183%
48	DSGb5 (d18:1/24:1) 1	1039.5 / 290.1	7249	21149	292%
49	DSGb5 (d18:1/24:1) 2	1039.5 / 290.1	69641	105932	152%
50	DSGb5 (d18:1/26:0) 1	1054.6 / 290.1	844	2604	309%
51	DSGb5 (d18:1/26:0) 2	1054.6 / 290.1	0	0	#DIV/0!
52	DSGb5 (d18:1/28:0)	1068.6 / 290.1	0	2331	#DIV/0!
53	DSGb5 (d18:1/30:0)	1082.6 / 290.1	3453	0	0%
54	GalNAcDSLc4, RM2 (d18:1/0:0)	885.9 / 290.1	0	1854	#DIV/0!
55	GalNAcDSLc4, RM2 (d18:1/16:0)	1005.0 / 290.1	102894	101828	99%
56	GalNAcDSLc4, RM2 (d18:1/18:0)	1019.0 / 290.1	1762	1538	87%
57	GalNAcDSLc4, RM2 (d18:1/18:1)	1018.0 / 290.1	3075	1496	49%
58	GalNAcDSLc4, RM2 (d18:1/18:2)	1017.0 / 290.1	1918	1152	60%
59	GalNAcDSLc4, RM2 (d18:1/18:3)	1016.0 / 290.1	766	0	0%
60	GalNAcDSLc4, RM2 (d18:1/20:0) 1	1033.0 / 290.1	7027	11165	159%

No.	Components	**Q1/Q3** **(*m/z*)**	**Medium HK-2** **(counts)**	**Medium ACHN** **(counts)**	**Ratio ACHN to HK-2** **(%)**
61	GalNAcDSLc4, RM2 (d18:1/20:0) 2	1033.0 / 290.1	1536	5028	327%
62	GalNAcDSLc4, RM2 (d18:1/20:1)	1032.0 / 290.1	5805	8399	145%
63	GalNAcDSLc4, RM2 (d18:1/20:2)	1031.0 / 290.1	2350	0	0%
64	GalNAcDSLc4, RM2 (d18:1/20:3)	1030.0 / 290.1	0	0	#DIV/0!
65	GalNAcDSLc4, RM2 (d18:1/20:4)	1029.0 / 290.1	0	1169	#DIV/0!
66	GalNAcDSLc4, RM2 (d18:1/20:5)	1028.0 / 290.1	4633	8015	173%
67	GalNAcDSLc4, RM2 (d18:1/22:0) 1	1047.0 / 290.1	8974	9205	103%
68	GalNAcDSLc4, RM2 (d18:1/22:0) 2	1047.0 / 290.1	0	0	#DIV/0!
69	GalNAcDSLc4, RM2 (d18:1/22:1)	1046.0 / 290.1	2739	0	0%
70	GalNAcDSLc4, RM2 (d18:1/22:2)	1045.0 / 290.1	0	3179	#DIV/0!
71	GalNAcDSLc4, RM2 (d18:1/22:3)	1044.0 / 290.1	1146	2297	200%
72	GalNAcDSLc4, RM2 (d18:1/22:4)	1043.0 / 290.1	1921	0	0%
73	GalNAcDSLc4, RM2 (d18:1/22:5) 1	1042.0 / 290.1	3097	2628	85%
74	GalNAcDSLc4, RM2 (d18:1/22:5) 2	1042.0 / 290.1	767	3510	458%
75	GalNAcDSLc4, RM2 (d18:1/22:6) 1	1041.0 / 290.1	18138	15055	83%
76	GalNAcDSLc4, RM2 (d18:1/22:6) 2	1041.0 / 290.1	17685	26047	147%
77	GalNAcDSLc4, RM2 (d18:1/24:0)	1061.1 / 290.1	3460	1153	33%
78	GalNAcDSLc4, RM2 (d18:1/24:1)	1060.1 / 290.1	24609	16633	68%
79	GalNAcDSLc4, RM2 (d18:1/26:0)	1075.1 / 290.1	0	1581	#DIV/0!
80	GalNAcDSLc4, RM2 (d18:1/28:0)	1089.1 / 290.1	13498	7862	58%

No.	Components	**Q1/Q3** **(*m/z*)**	**Medium HK-2** **(counts)**	**Medium ACHN** **(counts)**	**Ratio ACHN to HK-2** **(%)**
81	GalNAcDSLc4, RM2 (d18:1/30:0)	1103.1 / 290.1	109021	76312	70%
82	GD1a (d18:1/0:0)	784.3 / 290.1	0	0	#DIV/0!
83	GD1a (d18:1/16:0)	903.5 / 290.1	6356833	7462803	117%
84	GD1a (d18:1/18:0)	917.5 / 290.1	4503695	4343382	96%
85	GD1a (d18:1/18:1) 1	916.5 / 290.1	980157	908599	93%
86	GD1a (d18:1/18:1) 2	916.5 / 290.1	345542	303590	88%
87	GD1a (d18:1/18:1) 3	916.5 / 290.1	7368	6287	85%
88	GD1a (d18:1/18:2) 1	915.5 / 290.1	113897	107076	94%
89	GD1a (d18:1/18:2) 2	915.5 / 290.1	29779	17256	58%
90	GD1a (d18:1/18:3)	914.5 / 290.1	326274	339623	104%
91	GD1a (d18:1/20:0)	931.5 / 290.1	435727	353959	81%
92	GD1a (d18:1/20:1)	930.5 / 290.1	200184	161923	81%
93	GD1a (d18:1/20:2)	929.5 / 290.1	105557	89529	85%
94	GD1a (d18:1/20:3)	928.5 / 290.1	242421	243979	101%
95	GD1a (d18:1/20:4)	927.5 / 290.1	10876	15471	142%
96	GD1a (d18:1/20:5)	926.5 / 290.1	24006	20186	84%
97	GD1a (d18:1/22:0) 1	945.5 / 290.1	100730	47248	47%
98	GD1a (d18:1/22:0) 2	945.5 / 290.1	755516	527555	70%
99	GD1a (d18:1/22:1) 1	944.5 / 290.1	1533	52632	3433%
100	GD1a (d18:1/22:1) 2	944.5 / 290.1	252431	210954	84%

No.	Components	**Q1/Q3** **(*m/z*)**	**Medium HK-2** **(counts)**	**Medium ACHN** **(counts)**	**Ratio ACHN to HK-2** **(%)**
101	GD1a (d18:1/22:1) 3	944.5 / 290.1	27305	26315	96%
102	GD1a (d18:1/22:2)	943.5 / 290.1	1921	107521	5597%
103	GD1a (d18:1/22:3)	942.5 / 290.1	38029	45746	120%
104	GD1a (d18:1/22:4)	941.5 / 290.1	372966	387006	104%
105	GD1a (d18:1/22:5)	940.5 / 290.1	46955	44736	95%
106	GD1a (d18:1/22:6)	939.5 / 290.1	103889	103849	100%
107	GD1a (d18:1/24:0) 1	959.5 / 290.1	409893	322843	79%
108	GD1a (d18:1/24:0) 2	959.5 / 290.1	278215	186395	67%
109	GD1a (d18:1/24:1) 1	958.5 / 290.1	105079	67524	64%
110	GD1a (d18:1/24:1) 2	958.5 / 290.1	1089524	696064	64%
111	GD1a (d18:1/24:1) 3	958.5 / 290.1	14401	9265	64%
112	GD1a (d18:1/26:0) 1	973.5 / 290.1	4066	2260	56%
113	GD1a (d18:1/26:0) 2	973.5 / 290.1	2305	1993	86%
114	GD1a (d18:1/28:0)	987.6 / 290.1	6150	25197	410%
115	GD1a (d18:1/30:0)	1001.6 / 290.1	0	0	#DIV/0!
116	GM1 (d18:1/0:0)	1278.6 / 290.1	717	0	0%
117	GM1 (d18:1/16:0)	1516.8 / 290.1	18070	13072	72%
118	GM1 (d18:1/18:0)	1544.9 / 290.1	1922	6921	360%
119	GM1 (d18:1/18:1)	1542.9 / 290.1	3448	1170	34%
120	GM1 (d18:1/18:2)	1540.8 / 290.1	2691	1153	43%

No.	Components	**Q1/Q3 (*m/z*)**	**Medium HK-2 (counts)**	**Medium ACHN (counts)**	**Ratio ACHN to HK-2 (%)**
121	GM1 (d18:1/18:3)	1538.8 / 290.1	4614	1153	25%
122	GM1 (d18:1/20:0)	1572.9 / 290.1	0	0	#DIV/0!
123	GM1 (d18:1/20:1)	1570.9 / 290.1	0	0	#DIV/0!
124	GM1 (d18:1/20:2)	1568.9 / 290.1	0	1153	#DIV/0!
125	GM1 (d18:1/20:3)	1566.9 / 290.1	0	1153	#DIV/0!
126	GM1 (d18:1/20:4)	1564.8 / 290.1	0	0	#DIV/0!
127	GM1 (d18:1/20:5)	1562.8 / 290.1	769	0	0%
128	GM1 (d18:1/22:0)	1600.9 / 290.1	0	0	#DIV/0!
129	GM1 (d18:1/22:1)	1598.9 / 290.1	0	0	#DIV/0!
130	GM1 (d18:1/22:2)	1596.9 / 290.1	0	0	#DIV/0!
131	GM1 (d18:1/22:3)	1594.9 / 290.1	0	0	#DIV/0!
132	GM1 (d18:1/22:4)	1592.9 / 290.1	769	0	0%
133	GM1 (d18:1/22:5)	1590.9 / 290.1	0	0	#DIV/0!
134	GM1 (d18:1/22:6)	1588.8 / 290.1	0	0	#DIV/0!
135	GM1 (d18:1/24:0)	1629.0 / 290.1	1922	1933	101%
136	GM1 (d18:1/24:1)	1626.9 / 290.1	0	0	#DIV/0!
137	GM1 (d18:1/26:0)	1657.0 / 290.1	0	0	#DIV/0!
138	GM1 (d18:1/28:0)	1685.0 / 290.1	0	0	#DIV/0!
139	GM1 (d18:1/30:0)	1713.1 / 290.1	0	0	#DIV/0!
140	GM2 (d18:1/0:0)	1116.6 / 290.1	3844	1403	36%

No.	Components	**Q1/Q3** **(*m/z*)**	**Medium HK-2** **(counts)**	**Medium ACHN** **(counts)**	**Ratio ACHN to HK-2** **(%)**
141	GM2 (d18:1/16:0)	1354.8 / 290.1	21530	53059	246%
142	GM2 (d18:1/18:0)	1382.8 / 290.1	6536	5767	88%
143	GM2 (d18:1/18:1)	1380.8 / 290.1	769	0	0%
144	GM2 (d18:1/18:2)	1378.8 / 290.1	0	0	#DIV/0!
145	GM2 (d18:1/18:3)	1376.8 / 290.1	0	0	#DIV/0!
146	GM2 (d18:1/20:0)	1410.8 / 290.1	0	0	#DIV/0!
147	GM2 (d18:1/20:1)	1408.8 / 290.1	0	0	#DIV/0!
148	GM2 (d18:1/20:2)	1406.8 / 290.1	0	0	#DIV/0!
149	GM2 (d18:1/20:3)	1404.8 / 290.1	0	0	#DIV/0!
150	GM2 (d18:1/20:4)	1402.8 / 290.1	0	0	#DIV/0!
151	GM2 (d18:1/20:5)	1400.8 / 290.1	1158	769	66%
152	GM2 (d18:1/22:0)	1438.9 / 290.1	0	2307	#DIV/0!
153	GM2 (d18:1/22:1)	1436.9 / 290.1	0	0	#DIV/0!
154	GM2 (d18:1/22:2)	1434.8 / 290.1	0	1153	#DIV/0!
155	GM2 (d18:1/22:3)	1432.8 / 290.1	0	0	#DIV/0!
156	GM2 (d18:1/22:4)	1430.8 / 290.1	7715	6548	85%
157	GM2 (d18:1/22:5)	1428.8 / 290.1	21530	10767	50%
158	GM2 (d18:1/22:6)	1426.8 / 290.1	769	0	0%
159	GM2 (d18:1/24:0)	1466.9 / 290.1	3460	4229	122%
160	GM2 (d18:1/24:1)	1464.9 / 290.1	769	769	100%

No.	Components	**Q1/Q3 (*m/z*)**	**Medium HK-2 (counts)**	**Medium ACHN (counts)**	**Ratio ACHN to HK-2 (%)**
161	GM2 (d18:1/26:0)	1494.9 / 290.1	0	0	#DIV/0!
162	GM2 (d18:1/28:0)	1523.0 / 290.1	0	0	#DIV/0!
163	GM2 (d18:1/30:0)	1551.0 / 290.1	4231	1922	45%
164	GM3 (d18:1/0:0)	913.5 / 290.1	4927	1462	30%
165	GM3 (d18:1/16:0)	1151.7 / 290.1	6482229	5807321	90%
166	GM3 (d18:1/18:0)	1179.7 / 290.1	2156192	1774185	82%
167	GM3 (d18:1/18:1)	1177.7 / 290.1	687319	542265	79%
168	GM3 (d18:1/18:2)	1175.7 / 290.1	8060	0	0%
169	GM3 (d18:1/18:3)	1173.7 / 290.1	7597	8458	111%
170	GM3 (d18:1/20:0)	1207.8 / 290.1	144974	89604	62%
171	GM3 (d18:1/20:1)	1205.8 / 290.1	68052	37698	55%
172	GM3 (d18:1/20:2)	1203.7 / 290.1	59209	52619	89%
173	GM3 (d18:1/20:3)	1201.7 / 290.1	752	750	100%
174	GM3 (d18:1/20:4)	1199.7 / 290.1	3075	2690	87%
175	GM3 (d18:1/20:5)	1197.7 / 290.1	28225	12759	45%
176	GM3 (d18:1/22:0)	1235.8 / 290.1	252478	142274	56%
177	GM3 (d18:1/22:1)	1233.8 / 290.1	121510	56243	46%
178	GM3 (d18:1/22:2)	1231.8 / 290.1	18479	11919	65%
179	GM3 (d18:1/22:3)	1229.8 / 290.1	1537	0	0%
180	GM3 (d18:1/22:4)	1227.7 / 290.1	0	0	#DIV/0!

No.	Components	**Q1/Q3 (*m/z*)**	**Medium HK-2 (counts)**	**Medium ACHN (counts)**	**Ratio ACHN to HK-2 (%)**
181	GM3 (d18:1/22:5)	1225.7 / 290.1	8021	7546	94%
182	GM3 (d18:1/22:6)	1223.7 / 290.1	45318	25288	56%
183	GM3 (d18:1/24:0)	1263.8 / 290.1	595214	273110	46%
184	GM3 (d18:1/24:1)	1261.8 / 290.1	232662	96523	41%
185	GM3 (d18:1/26:0)	1291.9 / 290.1	3845	3460	90%
186	GM3 (d18:1/28:0)	1319.9 / 290.1	769	0	0%
187	GM3 (d18:1/30:0)	1347.9 / 290.1	0	769	#DIV/0!
188	MSGb5 (d18:1/0:0)	719.8 / 290.1	3319	11150	336%
189	MSGb5 (d18:1/16:0) 1	838.9 / 290.1	3012	14473	481%
190	MSGb5 (d18:1/16:0) 2	838.9 / 290.1	539356	1379809	256%
191	MSGb5 (d18:1/18:0) 1	853.0 / 290.1	4596	79439	1728%
192	MSGb5 (d18:1/18:0) 2	853.0 / 290.1	45099	65391	145%
193	MSGb5 (d18:1/18:1) 1	851.9 / 290.1	16477	18545	113%
194	MSGb5 (d18:1/18:1) 2	851.9 / 290.1	16477	20710	126%
195	MSGb5 (d18:1/18:2)	850.9 / 290.1	7304	13916	191%
196	MSGb5 (d18:1/18:3)	849.9 / 290.1	4523	1072	24%
197	MSGb5 (d18:1/20:0) 1	867.0 / 290.1	2340	4942	211%
198	MSGb5 (d18:1/20:0) 2	867.0 / 290.1	8043	9254	115%
199	MSGb5 (d18:1/20:1)	866.0 / 290.1	767	1061	138%
200	MSGb5 (d18:1/20:2) 1	865.0 / 290.1	0	3911	#DIV/0!

No.	Components	**Q1/Q3** **(*m/z*)**	**Medium HK-2** **(counts)**	**Medium ACHN** **(counts)**	**Ratio ACHN to HK-2** **(%)**
201	MSGb5 (d18:1/20:2) 2	865.0 / 290.1	9227	6114	66%
202	MSGb5 (d18:1/20:3) 1	863.9 / 290.1	3755	11448	305%
203	MSGb5 (d18:1/20:3) 2	863.9 / 290.1	13443	10449	78%
204	MSGb5 (d18:1/20:4)	862.9 / 290.1	2293	8648	377%
205	MSGb5 (d18:1/20:5)	861.9 / 290.1	1426	1171	82%
206	MSGb5 (d18:1/22:0) 1	881.0 / 290.1	1982	3118	157%
207	MSGb5 (d18:1/22:0) 2	881.0 / 290.1	22839	32296	141%
208	MSGb5 (d18:1/22:1) 1	880.0 / 290.1	8715	12298	141%
209	MSGb5 (d18:1/22:1) 2	880.0 / 290.1	1918	1538	80%
210	MSGb5 (d18:1/22:2)	879.0 / 290.1	4736	4526	96%
211	MSGb5 (d18:1/22:3) 1	878.0 / 290.1	13393	8894	66%
212	MSGb5 (d18:1/22:3) 2	878.0 / 290.1	744	2252	303%
213	MSGb5 (d18:1/22:4)	877.0 / 290.1	3106	6557	211%
214	MSGb5 (d18:1/22:5)	875.9 / 290.1	759	1317	174%
215	MSGb5 (d18:1/22:6)	874.9 / 290.1	0	4225	#DIV/0!
216	MSGb5 (d18:1/24:0) 1	895.0 / 290.1	16116	17922	111%
217	MSGb5 (d18:1/24:0) 2	895.0 / 290.1	41131	58437	142%
218	MSGb5 (d18:1/24:1) 1	894.0 / 290.1	1123	3228	287%
219	MSGb5 (d18:1/24:1) 2	894.0 / 290.1	58917	56135	95%
220	MSGb5 (d18:1/24:1) 3	894.0 / 290.1	2304	1923	83%

No.	Components	**Q1/Q3** **(*m/z*)**	**Medium HK-2** **(counts)**	**Medium ACHN** **(counts)**	**Ratio ACHN to HK-2** **(%)**
221	MSGb5 (d18:1/26:0)	909.0 / 290.1	0	5915	#DIV/0!
222	MSGb5 (d18:1/28:0)	923.0 / 290.1	67320	64147	95%
223	MSGb5 (d18:1/30:0)	937.1 / 290.1	13386	13134	98%
224	Speculated 1 (d18:1/0:0) 1	946.4 / 290.1	438591	333248	76%
225	Speculated 1 (d18:1/0:0) 2	946.4 / 290.1	33455	28267	84%
226	Speculated 1 (d18:1/16:0)	1065.5 / 290.1	0	0	#DIV/0!
227	Speculated 1 (d18:1/18:0)	1079.5 / 290.1	5690	5766	101%
228	Speculated 1 (d18:1/18:1)	1078.5 / 290.1	36236	26488	73%
229	Speculated 1 (d18:1/18:2)	1077.5 / 290.1	104499	147962	142%
230	Speculated 1 (d18:1/18:3)	1076.5 / 290.1	74024	70640	95%
231	Speculated 1 (d18:1/20:0)	1093.5 / 290.1	3707	3710	100%
232	Speculated 1 (d18:1/20:1)	1092.5 / 290.1	41831	26670	64%
233	Speculated 1 (d18:1/20:2)	1091.5 / 290.1	206421	159071	77%
234	Speculated 1 (d18:1/20:3)	1090.5 / 290.1	106791	74478	70%
235	Speculated 1 (d18:1/20:4)	1089.5 / 290.1	38061	29185	77%
236	Speculated 1 (d18:1/20:5)	1088.5 / 290.1	10669	12599	118%
237	Speculated 1 (d18:1/22:0)	1107.6 / 290.1	2671	1907	71%
238	Speculated 1 (d18:1/22:1)	1106.6 / 290.1	15721	7612	48%
239	Speculated 1 (d18:1/22:2)	1105.5 / 290.1	68711	55224	80%
240	Speculated 1 (d18:1/22:3) 1	1104.5 / 290.1	363038	269914	74%

No.	Components	**Q1/Q3 (*m/z*)**	**Medium HK-2 (counts)**	**Medium ACHN (counts)**	**Ratio ACHN to HK-2 (%)**
241	Speculated 1 (d18:1/22:3) 2	1104.5 / 290.1	40731	32084	79%
242	Speculated 1 (d18:1/22:4)	1103.5 / 290.1	183867	132069	72%
243	Speculated 1 (d18:1/22:5)	1102.5 / 290.1	43651	27574	63%
244	Speculated 1 (d18:1/22:6)	1101.5 / 290.1	6108	7191	118%
245	Speculated 1 (d18:1/24:0)	1121.6 / 290.1	15660	19960	127%
246	Speculated 1 (d18:1/24:1)	1120.6 / 290.1	0	1927	#DIV/0!
247	Speculated 1 (d18:1/26:0)	1135.6 / 290.1	777	1137	146%
248	Speculated 1 (d18:1/28:0)	1149.6 / 290.1	430305	378901	88%
249	Speculated 1 (d18:1/30:0)	1163.6 / 290.1	1152	7443	646%
250	Speculated 2 (d18:1/0:0) 1	966.9 / 290.1	104210	66721	64%
251	Speculated 2 (d18:1/0:0) 2	966.9 / 290.1	7956	15874	200%
252	Speculated 2 (d18:1/16:0)	1086.0 / 290.1	39957	44680	112%
253	Speculated 2 (d18:1/18:0)	1100.0 / 290.1	19941	10657	53%
254	Speculated 2 (d18:1/18:1)	1099.0 / 290.1	45273	22103	49%
255	Speculated 2 (d18:1/18:2)	1098.0 / 290.1	28004	16338	58%
256	Speculated 2 (d18:1/18:3)	1097.0 / 290.1	42198	33681	80%
257	Speculated 2 (d18:1/20:0)	1114.1 / 290.1	5927	5899	100%
258	Speculated 2 (d18:1/20:1)	1113.1 / 290.1	33366	21842	65%
259	Speculated 2 (d18:1/20:2)	1112.0 / 290.1	63137	54166	86%
260	Speculated 2 (d18:1/20:3)	1111.0 / 290.1	4052	7439	184%

No.	Components	**Q1/Q3** **(*m/z*)**	**Medium HK-2** **(counts)**	**Medium ACHN** **(counts)**	**Ratio ACHN to HK-2** **(%)**
261	Speculated 2 (d18:1/20:4)	1110.0 / 290.1	4739	6411	135%
262	Speculated 2 (d18:1/20:5)	1109.0 / 290.1	5427	7379	136%
263	Speculated 2 (d18:1/22:0)	1128.1 / 290.1	2691	697	26%
264	Speculated 2 (d18:1/22:1)	1127.1 / 290.1	5039	4755	94%
265	Speculated 2 (d18:1/22:2)	1126.1 / 290.1	11133	3393	30%
266	Speculated 2 (d18:1/22:3) 1	1125.1 / 290.1	34650	23678	68%
267	Speculated 2 (d18:1/22:3) 2	1125.1 / 290.1	54594	53640	98%
268	Speculated 2 (d18:1/22:4)	1124.0 / 290.1	233796	241133	103%
269	Speculated 2 (d18:1/22:5)	1123.0 / 290.1	145890	162177	111%
270	Speculated 2 (d18:1/22:6)	1122.0 / 290.1	10967	12605	115%
271	Speculated 2 (d18:1/24:0)	1142.1 / 290.1	0	0	#DIV/0!
272	Speculated 2 (d18:1/24:1)	1141.1 / 290.1	3873	4104	106%
273	Speculated 2 (d18:1/26:0)	1156.1 / 290.1	21137	13065	62%
274	Speculated 2 (d18:1/28:0)	1170.1 / 290.1	116521	82862	71%
275	Speculated 2 (d18:1/30:0)	1184.1 / 290.1	3429	3074	90%
276	Total		34269386	36805553	107%
