# Supplementary material for: Wide-Targeted Semi-Quantitative Analysis of Acidic Glycosphingolipids in Cell Lines and Urine to Develop Potential Screening Biomarkers for Renal Cell Carcinoma
Source: Int J Mol Sci. 2024 Apr 7;25(7):4098. doi: 10.3390/ijms25074098 (PMC11012862; doi:10.3390/ijms25074098)
Supplement: Supplementary file 1 [file ijms-25-04098-s001.zip › TableS6_2.0.pdf]

Table S6. Summary of GSL peaks detected in the cell lines.

(a) Peak number of GSLs in cells.

	Not detected (n)	Detected (n)
HK-2	52	223
ACHN	2	273

(b) Peak number of GSLs in the medium.

	Not detected (n)	Detected (n)
HK-2	71	204
ACHN	68	207

(c) Peak number of changes between HK-2 and ACHN media.

	Cells (n)	Medium (n)
Decreased	3	121
Unchanged	0	1
Increased	223	82

Decreased means the peak ratio of GSLs in ACHN/HK-2 less than 100%, “Unchanged” means the peak ratio of GSLs in ACHN/HK-2 100%, “Increased” means the peak ratio of GSLs in ACHN/HK-2 more than 100%.

(d) Intense peak number of GSLs in the medium.

	The peak with more than 100,000 counts/cells (n)	The peak with more than 2,000,000 counts/cells (n)
HK-2	42	4
ACHN	36	4
