# Supplementary material for: Wide-Targeted Semi-Quantitative Analysis of Acidic Glycosphingolipids in Cell Lines and Urine to Develop Potential Screening Biomarkers for Renal Cell Carcinoma
Source: Int J Mol Sci. 2024 Apr 7;25(7):4098. doi: 10.3390/ijms25074098 (PMC11012862; doi:10.3390/ijms25074098)
Supplement: Supplementary file 1 [file ijms-25-04098-s001.zip › TableS7_2.0.pdf]

Table S7 Urinary peak intensity of DSGb5 (d18:1/16:0).

No	Group	Sample	SEX	Age (years)	Urinary DSGb5 (d18:1/16:0) (Counts/mg creatinine)
1	Ctrl	Ctrl01	Male	21	1236
2	Ctrl	Ctrl02	Male	22	966
3	Ctrl	Ctrl03	Male	23	1193
4	Ctrl	Ctrl04	Female	44	4002
5	Ctrl	Ctrl05	Male	29	4864
6	Ctrl	Ctrl06	Male	47	2161
7	Ctrl	Ctrl07	Male	31	3573
8	Ctrl	Ctrl08	Male	36	9274
9	Ctrl	Ctrl09	Male	36	2083
10	RCC	RCC01	Male	73	8407
11	RCC	RCC02	Male	58	25472
12	RCC	RCC03	Male	60	9435
13	RCC	RCC04	Male	50	9654
14	RCC	RCC05	Male	48	18486
15	RCC	RCC06	Male	57	53786
16	RCC	RCC07	Female	51	16928
17	RCC	RCC08	Male	79	12202
18	RCC	RCC09	Female	65	8653
19	RCC	RCC10	Male	81	14864
20	RCC	RCC11	Male	54	11437
21	RCC	RCC12	Male	48	447
22	RCC	RCC13	Male	34	3572
23	RCC	RCC14	Female	63	49332
24	RCC	RCC15	Male	56	49638
