# Supplementary material for: Wide-Targeted Semi-Quantitative Analysis of Acidic Glycosphingolipids in Cell Lines and Urine to Develop Potential Screening Biomarkers for Renal Cell Carcinoma
Source: Int J Mol Sci. 2024 Apr 7;25(7):4098. doi: 10.3390/ijms25074098 (PMC11012862; doi:10.3390/ijms25074098)
Supplement: Supplementary file 1 [file ijms-25-04098-s001.zip › TableS8_2.0.pdf]

Table S8 Demographics of healthy controls and RCC patients.

Group	Healthy controls	RCC patients	*P* values
Number of samples (n)	9	15	
Male (%)	11.1	20.0	0.5716
Mean ± SD (years)	32.1 ± 3.82	58.5 ± 2.96	<0.001

RCC, renal cell carcinoma.
